# Decomposing user-defined tasks in a reinforcement learning setup using TextWorld

**DOI:** 10.3389/frobt.2023.1280578

**Published:** 2023-12-22

**Authors:** Thanos Petsanis, Christoforos Keroglou, Athanasios Ch. Kapoutsis, Elias B. Kosmatopoulos, Georgios Ch. Sirakoulis

**Affiliations:** ^1^ School of Engineering, Department of Electrical and Computer Engineering, Democritus University of Thrace (DUTH), Xanthi, Greece; ^2^ The Centre for Research and Technology, Information Technologies Institute, Thessaloniki, Greece

**Keywords:** formal methods in robotics and automation, reinforcement learning, hierarchical reinforcement learning, task and motion planning, autonomous agents

## Abstract

The current paper proposes a hierarchical reinforcement learning (HRL) method to decompose a complex task into simpler sub-tasks and leverage those to improve the training of an autonomous agent in a simulated environment. For practical reasons (i.e., illustrating purposes, easy implementation, user-friendly interface, and useful functionalities), we employ two Python frameworks called TextWorld and MiniGrid. MiniGrid functions as a 2D simulated representation of the real environment, while TextWorld functions as a high-level abstraction of this simulated environment. Training on this abstraction disentangles manipulation from navigation actions and allows us to design a dense reward function instead of a sparse reward function for the lower-level environment, which, as we show, improves the performance of training. Formal methods are utilized throughout the paper to establish that our algorithm is not prevented from deriving solutions.

## 1 Introduction

In recent years, there has been a surge of effort and resources invested into what was dubbed “Embodied AI” (Yenamandra et al., 2023b; a; Gao et al., 2022; Duan et al., 2022), which is described as robotic agents that have the ability to exist in the real world (have a body), learn from it, and interact with it. The overall goal of this field of AI is to deliver robotic agents that carry out tasks, manipulate objects, and can serve people in their daily routines. There are multitudes of challenges arising from this field, such as navigation (Gervet et al., 2022), natural language processing (NLP) (Shridhar et al., 2020a), simulation (Weihs et al., 2020), computer vision (Anderson et al., 2018), motion and task planning (Garrett et al., 2021), manipulation challenges (Xie et al., 2019), challenges involving all of these (Bohren et al., 2011), and even hardware limitations, just to name a few. However, another perhaps often undervalued challenge is breaking down the inherently complex language commands into simpler, more manageable, sub-commands or “sub-tasks.” This is partly an NLP challenge and, at the same time, a task planning challenge.

### 1.1 Problem statement

Let us consider a case where the user issues a command to the robotic agent in the form of natural language. This voice command can easily be converted into text for the agent to process textual data rather than sound. The agent would then need to map this text command into robotic commands in order to execute it. The command given is not based on a standardized set of commands (e.g., “move forward” and “turn right”) but, instead, is based on the common tongue and human understanding. This means that depending on the complexity of the command, mapping it into robotic actuation can prove a difficult objective. For example, commands such as “grab the water bottle in front of you” is a relatively simple task that, given proper NLP, involves a computer vision task requiring to recognize the object “water bottle” currently within the agent’s vision and a manipulation task requiring to grab it. Relatively, that is, to commands such as “bring the water bottle” do not clarify where the water bottle is. If not currently within vision, the agent would need to search for it, adding the task of navigation. Additionally, if there was a door or obstacle in the way of navigation, another manipulation task would result from the command.

Conventionally, the mapping of the user command to robotic actuations that the different tasks are comprised is done within a neural network (NN) which is trained via reinforcement learning (RL)^1^. The NN, given the input, the user command, and the state it is currently in, would output the actions to perform in the current time step. If the actions performed led to a positive reward, the weights of the network would be altered in a way that “reinforces” those actions. In other words, as long as we can map the user commands to rewards for specific goal states, NN will learn the mapping of its input (state + user command) to its output (current actions to take) in order to reach those goal states. For example, considering the previous command “bring the water bottle,” the goal state would be the agent holding the water bottle in front of the person who asked. Automatically assigning rewards to goal states based on the user command is one problem. However, despite assuming we have a module that does this, there is still the problem of sparse rewarding remaining, i.e., before the NN is trained, it takes random actions (exploration), and due to the complexity of the environment, it might never (or very rarely) reach the goal-only sparse reward and, therefore, might never (or very poorly) learn the correct actions. Furthermore, it is important to note that, in our workspace, in most simulations and in the real world, the agent needs to have the right orientation and location before choosing the right pick/place or open/close action. Therefore, even a simple space of 6 × 6 cells in a grid environment (see Figure 4) can lead to a high number of reward-less actions before the agent is rewarded, which can result in extreme slow learning. Overall, robotic tasks tend to prove hard in terms of constructing a reward function due to complex state space representations and usually demand a human-in-the-loop approach (Singh et al., 2019). This has been well-documented in the literature (Kober et al., 2013) and results in sparse rewarding (Rauber et al., 2021; Rengarajan et al., 2022) or even the binary goal reward (1 if the goal state is reached and 0 otherwise). The same is true in the case of embodied AI. Hence, dense rewarding is desirable. We show that reward assignment in such cases can be simplified with task decomposition by implementing an approach that tackles these problems. Using formal methods, we guarantee it can reach solutions, and we present proof-of-concept examples and environment testing.

We argue that TextWorld is a Python framework that can deal with the aforementioned challenges (Côté et al., 2019). Briefly, TextWorld is an open-source text-based game developed by Microsoft and aims to assist the development of NLP agents. In TextWorld, every “world” is described in text form (hence, the name), and the player can provide written commands to navigate this world. The goal of the game is also described as text and often entails pick-and-place objectives. Every environment has a goal defined as a series of actions being taken where each action is a string. We take advantage of the useful functionalities of TextWorld, such as language understanding, textual representation of a state, and most notably, an extendable knowledge base. The knowledge base is a set of rules that is applied to the environments. It means that we have some *a priori* knowledge about the context of every environment and the “meaningful” interactions with the objects that can be found in TextWorld. This knowledge contains information about a limited amount of actions that are sensible to take (e.g., “grab the water bottle”) and excludes the rest (e.g., “eat the water bottle”), which is pivotal to encode in our RL setup. Essentially, the knowledge base reflects common sense reasoning, which is necessary when we have object interaction and we cannot afford computational resources to explore all possible combinations of legal actions with existing objects. Moreover, it contains sufficient information about the different ways of describing the same command (e.g., “put the water bottle on the table” and “place the bottle of water on the table” are the same commands written differently)^2^. Afterward, the Python framework we chose as the simulator for testing our method is MiniGrid. MiniGrid is a 2D grid-world simulation that can be considered an abstraction of the real environment. It is simple enough compared to TextWorld that it only adds navigation actions to its complexity and has very similar object interactions to it. Consequently, it not only leaves room for lower levels of hierarchy (e.g., MiniWorld Chevalier-Boisvert, 2018) but also coherently proceeds with the higher level of our hierarchy.

Our approach and embodied AI research, in general, can have great implications in the flourishing field of human–machine collaboration and, more specifically, industrial robotics. According to the flowchart drawn by Konstantinidis et al. (2022), it could lead to business opportunities (and we have already seen examples of robot assistant products or pets (Keroglou et al. 2023), research opportunities (as we have already seen advancement in RL research that answers the high demands of robotic agents like massive datasets in Deitke et al. (2022) and competitions), and lastly, the next human-centered industrial revolution called “Industry 5.0” (that emphasizes human–machine interaction). Othman et al. (2016) also shows the use of robots in advanced manufacturing systems that will, no doubt, be benefited by embodied AI research.

### 1.2 Related work

Our method is inspired by ideas in the areas of reward engineering (Ng et al. (1999); Laud (2004); Toro Icarte et al. (2020)), hierarchical reinforcement learning (HRL) (Dietterich et al. (1998); Barto and Mahadevan (2003)), dense rewarding of sub-tasks (Xie et al., 2019), and, most notably, from another TextWorld implementation called ALFWorld (Shridhar et al., 2020b). To avoid any confusion between these two similar works, we highlight the differences and concurrently our contributions. In ALFWorld, the BUTLER agent also incorporates TextWorld inside its framework in order to learn abstract high-level actions before translating them to low-level actions (inside the ALFRED-simulated environment). Both works first train a TextWorld agent. The key difference lies in the translation between TextWorld and the real problem. In other words, ALFWorld directly translates the text–action output of the agent to simulator actions, whereas we use the text–action outputs of our agent to embed more rewards inside the simulator and afterwards train a different low-level agent inside this now reward-dense simulator. Since this approach enriches the simulated environment, it is independent from the ML method and, thus, can be more wildly adopted. Normally, an environment should already possess reward-dense qualities to promote learning, but to the best of our knowledge, a lot of embodied AI challenges lack these dense reward functions. For example, in the study by Yadav et al. (2023), the distance-from-goal and a Boolean, indicating whether the goal has been reached or not, are provided and can be used as rewards, but it lacks sub-task rewards such as when obstacles are avoided and when a room in the right sequence of rooms is reached. In the recent Open-Vocabulary Mobile Manipulation (OVMM) challenge (Yenamandra et al., 2023b), competitors can define the pick reward and place reward for the pick-and-place tasks, but the challenge could still benefit from more reward definitions such as the distance-from-pick and distance-from-place rewards and rewards for “go to {room}” and “open/close door” commands (the last two are possible in our setup). Perhaps this happens because optimal action sequences (which would be the rewarded sub-actions) to reach the required goal cannot easily be defined. Furthermore, sub-optimal solutions should also be provided corresponding rewards, which increases the complexity. Another key difference is that we developed a holistic RL solution, meaning that low-level navigation and manipulation actions are also learned through training in an RL setup, whereas in ALFWorld, BUTLER uses the A* algorithm for navigation, pre-trained models for object segmentation, and other built-in algorithms for manipulation. Let us clarify here that the manipulation actions taken by the RL agent in our setup are of a higher level of hierarchy compared to ALFWorld (as for the navigation actions, they are quite similar). When we execute the manipulation action “pick up the bottle,” as long as the agent is next to the bottle and faces it, then the bottle is placed inside the agent’s inventory, whereas in ALFWorld, the agent executes relative arm-movement actions before interacting with the object. Despite this difference, RL agents are still preferred over standardized algorithms since they can be trained to perform tasks the algorithms would fail, either due to reaching their limits or because they are made to deal with a subset of problems.

During the development of this work, new technologies surfaced that are important to highlight and which could be beneficially combined with our approach. Reliable large language models (LLMs) such as ChatGPT (Liu et al., 2023) have emerged which can reason human speech. It could be argued that ChatGPT’s language understanding power and API can assist our current “decomposer” (i.e., TextWorld). Since TextWorld is a text-based description game, ChatGPT could quickly and reliably deduce the right actions and greatly decrease the exploration needed inside the TextWorld-dedicated RL agent. In short, it could be used as an expert demonstrator. In another example, TaskLAMA (Yuan et al., 2023) achieves general task decomposition via LLMs, but these modules could not replace TextWorld since they would first need an understanding/description of the environment the tasks are based upon. 3D LLMs (Hong et al., 2023) achieve just this. In other words, they extract textual descriptions from 3D features and can perform task decomposition. However, the researchers did not use the sub-tasks for enriching the simulations with rewards, and this is where our novelty lies. Other times, researchers will often manually decompose the problem, using their intuition, into sub-tasks and construct dedicated rewards in order to improve training, as was done by Kim et al. (2023), who split the pick-and-place task into approaching the object location, reaching the object position and grasping it. The current work is an HRL algorithm that performs the following tasks:

1. Decomposes a complex command to its simpler components;
2. Automatically integrates those sub-commands into environment rewards;
3. Learns to solve the reward-dense environment.

Similar ideas are used in the area of integration of task and motion planning (ITMP). For example, a high-level planner is presented by He et al. (2015), who solved a motion planning problem using a framework with three layers (high-level planner, coordinating layer, and low-level planner) but without applying reinforcement learning techniques.

The mathematical framework of our work is based on the implementation of formal methods. Formal methods were only recently studied in reinforcement learning setups, aiming to provide guarantees related to the behavior of the agent (Li et al., 2016; Alshiekh et al., 2018; Icarte et al., 2018). A popular line of work involves formal methods to provide safety guarantees. For example, Alshiekh et al. (2018) introduced the concept of “shielded reinforcement learning.” Specifically, they introduced safety rules expressed as finite-state machines. Using formal methods, we prove that in every case scenario, under generic and common assumptions (Definition 4), our algorithm is not constrained to derive solutions (Theorem 1). For practical reasons, we define and prove Theorem 1 by appropriately implementing two specific environments: a) a simulated environment (i.e., MiniGrid) and b) an abstraction of this environment (i.e., TextWorld). However, Theorem 1 can be proved true for any two environments (where the one is an abstraction of the other), which can be modeled as deterministic finite automata (DFA) (Keroglou and Dimarogonas, 2020a).

We aim to present a solution that utilizes the TextWorld framework to enhance the training in a simulated real-world environment for embodied AI. Our current work demonstrates the efficacy of the aforementioned solution in the simulated environment of MiniGrid. This work does not enhance simulations currently used in embodied AI such as Habitat (Manolis Savva* et al. (2019); RoboTHOR (2020)), and DialFRED (Gao et al., 2022). Instead, it serves as preliminary grounds to showcase the benefits of adopting it and to enable such extensions. The problem domain we address is sparse rewards in embodied AI simulations by employing complex task decomposition. In summary, our contributions are as follows:

1. An RL module that decomposes complex text commands into simpler sub-commands.
2. A holistic RL solution that empirically shows the advantages of dense-reward environments by leveraging the aforementioned contribution.
3. Proof, based on formal methods, that there exists a solution for our methodology.

All in all, with this paper, we first discussed the necessity of adequately solving sparse RL problems, then we propose an automatic and mathematically rigorous way of dividing the task at hand into “solvable” sub-tasks that will guide rewards, and, finally, we empirically demonstrate the performance improvements in an appropriate RL environment within a simulation framework. The paper is developed as follows: Section 2 presents important mathematical notions needed for the development of the material presented in the following sections and formally describes our methodology in detail; and Section 3 presents simulations 1) illustrating our method to a running example and 2) comparing our RL setup guided by a dense reward function that exploits task decomposition, with an RL setup guided by a simple reward function (without task decomposition). Finally, possible future extensions of our work are discussed in Section 4.

## 2 Materials and methods

**FIGURE 1:**
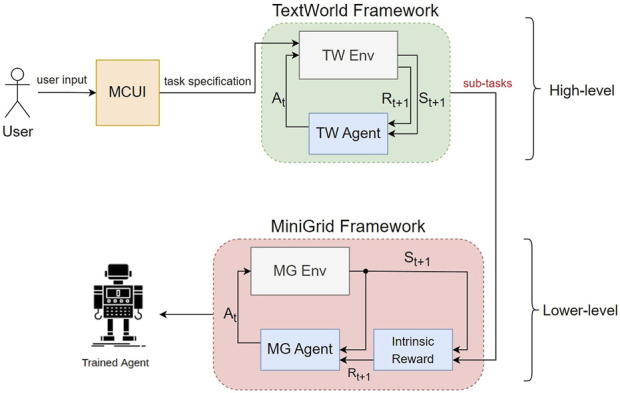
The proposed concept consists of i) a multiple choice UI (MCUI) which captures a user-defined task, ii) the TextWorld framework which is used to decompose the user-defined task into a set of simpler sub-tasks, and feeds iii) an RL setup in a simulated environment (in our example, MiniGrid) with an appropriate reward function.

In this section, we develop our methodology. First, we revisit the appropriate mathematical definitions (i.e., Markov decision processes (MDPs) in RL setups and finite-state automata) that are essential for understanding the TextWorld and MiniGrid automata. Later, we define the DFAs that describe our two simulation modules and briefly define the multiple choice user interface (MCUI) we constructed. These definitions summarize the essence of our methodology which we use to define the RL problem with mathematical notations. Finally, we prove that, using our method, the agent will not reach terminal states that are not goal states, and thus, a solution always exists. First, our objective is to train an agent capable of finding the solution of a complex user-defined task using reinforcement learning techniques. Part of the solution involves the automatic optimal decomposition of the complex task given by a user to a set of simpler sub-tasks that the agent needs to sequentially satisfy. We formally describe our solution as follows (see Figure 1 for the corresponding diagram):

1. An MCUI is implemented to express the user-defined task (see Section 2.5).
2. An abstraction of the workspace (of MiniGrid) is defined in TextWorld (see Section 2.4).
3. An RL problem (RL–TextWorld) is formulated with a user-defined task interpreted as a goal state (see Section 2.6).
4. An RL–MiniGrid problem (see Section 2.7) is formulated with a dense reward function derived from the solution of RL–TextWorld.
5. The performance of a PPO (Schulman et al., 2017) agent that solves the RL–MiniGrid problem is evaluated for the cases of dense and sparse (i.e., without exploiting the structure of the complex task) reward functions.

The following should be noted: the command requested as input by the user is a pick-and-place type command and must be in the specific form described in Section 2.5. The user can pick from a list of available items inside the house setting that are displayed in text form via MCUI before training starts. The TextWorld environment must be an accurate abstraction of the simulated environment. Dense rewards are embedded automatically to MiniGrid, given the TextWorld output.

Hereafter, we use the abbreviations “TW” and “MG” for the words TextWorld and MiniGrid, respectively. The entirety of our code can be found in GitHub^3^, as well as a notebook file which allows anyone to reproduce the results shown in Section 3 with Google Colab^4^.

### 2.1 Markov decision process of an RL setup

In a reinforcement learning setup, an agent is guided by appropriate rewards in order to learn a policy that maximizes the total return. In such a setup, the environment can often be modeled by an infinite Markov decision process, which is formally defined as follows.

Definition 1 *Markov decision process* (Li et al. 2016). *An infinite MDP is a tuple* (*S*, *A*, *p*(., ., .), *R*(.))*, where*

•*S* ⊆ *R* ^*n*^ *is a continuous set of states;*
•*A* ⊆ *R* ^*m*^ *is a continuous set of actions;*
•*p*: *S* × *A* × *S* → [0, 1] *is the transition probability function with* *p*(*s*, *a*, *s*′) *being the probability of taking action* *a* ∈ *A* *at state* *s* ∈ *S* *and ending up in state* *s*′ ∈ *S* (*also commonly written as a condition probability* *p*(*s*′ |*s*, *a*);
•*R:* *τ* → **R** *is the reward function which is defined in general for state–action trajectory* *τ* = (*s*_0_, *a*_0_, … , *s*_*T*_)*, where* *T* *is the finite horizon (i.e., maximum number of finite steps).*

The goal of a reinforcement learning problem is to find an optimal stochastic policy π*: *S* × *A* → [0, 1] that maximizes the expected accumulated reward, (i.e., $\pi^{*}=\arg\max_{\pi }\left(E_{p^{\pi }\left(\tau \right)}\left[R\left(\tau \right)\right]\right)$ , where *p* ^*π*^(*τ*) is the trajectory distribution from following policy *π*, and *R*(*τ*) is the reward obtained, given *τ*). The transition *p*(*s*, *a*, *s*′) is usually unknown to the learning agent.

### 2.2 Finite state automaton (FSA)

In the case studied in this paper (TW and MG environments), there is no uncertainty regarding the outcome of the executed action (deterministic setting). This allows us to express the workspace, along with the dynamics of the agent as a finite state automaton (FSA) (Keroglou and Dimarogonas, 2020b) and, in particular, as a deterministic finite automaton (DFA), which is defined as follows:

Definition 2 *Deterministic Finite Automaton (DFA). An FSA is captured by* *G* = (*X*, *Σ*, *δ*, *x*_0_, *F*)*, where*

•*X* = {1, 2, … , *N*} *is the set of states;*
•Σ *is the set of events (i.e., actions);*
•*δ*: *X* × Σ → 2^*X*^ *is, in general, a nondeterministic state transition function, and FSA is called a nondeterministic finite automaton (NFA). In the simpler case, where* *δ*: *X* ×Σ → *X* *, we call it a deterministic transition function, and FSA is called a deterministic finite automaton or DFA*. *For a set* *Q* ⊆ *X* *and* *σ* ∈ *Σ* *, we define* *δ*(*Q*, *σ*) = ⋃_*q*∈*Q*_ *δ*(*q*, *σ*). *Function* *δ* *can be extended from the domain* *X* ×Σ *to the domain* *X* ×Σ* *in a recursive manner:* *δ*(*x*, *σs*)≔*δ*(*δ*(*x*, *σ*), *s*) *for* *x* ∈ *X* *,* *s* ∈ Σ*, *and* *σ* ∈ Σ (*note that* *δ*(*x*, *ɛ*)≔{*x*});
•*x*_0_ *is the initial state; and*
•*F* *is the set of accept states.*

### 2.3 Simulated environment expressed in MiniGrid

Definition 3 *The simulated environment is captured in MiniGrid (grid workspace) by a DFA* *G* = (*X*_*g*_, Σ_*g*_, *δ*_*g*_, *X*_0,*g*_, *F*_*g*_)*, where*

•*X*_*g*_= {*p*_*a*_, *o*_1_, … , *o*_*n*_} *is the set of states, where*
  •*p*_*a*_= (*w*_*a*_, *h*_*a*_)is the position of the agent expressed as the cell (*w*_*a*_, *h*_*a*_) in an *M*× *N*grid, where 1 ≤ *w*_*a*_≤ *M*, 1 ≤ *h*_*a*_≤ *N*, and
  •*o*_*i*_= (*p*_*i*_, *c*_*i*_)are the properties of the object *obj*_*i*_∈ *O*, *O*= {*obj*_1_, … , *obj*_*n*_}, where
    – *p*_*i*_is the position of *obj*_*i*_and
    – *c*_*i*_= (*carry*(*i*), *type*(*i*), *prop*(*i*))is the configuration of *obj*_*i*_. The elements of *c*_*i*_are
      ∗*carry*(*i*),a function that indicates if the object is carried by the agent;
      ∗*type*(*i*)that indicates the type of the object (1 for movable objects, 2 for containers, and 3 for support objects); and
      ∗*prop*(*i*that indicates if the object is open or closed (*prop*(*i*) = 1or *prop*(*i*) = 0, respectively). For all objects that we cannot open or close, *prop*(*i*) = 0

• Σ_*g*_ = Σ_*N*,*g*_ ∪ Σ_*M*,*g*_ *is the set of actions, where*
• Σ_*N*,*g*_ = {*turn right*, *turn left*, *move forward*} is the set of navigation actions for *G*; and
• Σ_*M*,*g*_ = {*pick*, *drop*, *toggle*} is the set of manipulation actions for *G*
• *δ*_*g*_: *X*_*g*_ ×Σ_*g*_ → *X*_*g*_ *is the deterministic transition function.*
• *x*_0,*g*_ *is the initial state and*
• *F*_*g*_ = *X*_*g*_ *is the set of A states* (*i.e., the goal-states we are attempting to reach*)*.*

Definition 4 *We assume the following for the simulated environment in MiniGrid. Assumptions A1–A4:*

*A1*) *All objects (movable, support, and containers), rooms, doors, and connections between rooms are already identified.*
*A2*) *A room* *r* *is mapped to a set of cells in MiniGrid* $A_{r}=\left\{\left(w_{1,r},h_{1,r}\right),\ldots ,\left(w_{m_{r},r},h_{n_{r},r}\right)\right\}$ *, where for* 1 ≤ *i* ≤ *m*_*r*_ *,* 1 ≤ *j* ≤ *n*_*r*_ (*w*_*i*,*r*_, *h*_*j*,*r*_) *is a cell in the* *M* × *N* *grid. We also define the set* *A*_*r*,*f*_ ⊆ *A*_*r*_ *of all free cells that belong to a room as the cells where no support objects are placed.*
*A3*) *All free cells* (*A*_*r*,*f*_) *that belong to the same room are connected. This is formally defined as follows: for all objects, for any legal object configuration* *c*_*i*_ *, and for any pair of states* *x*_*i*_ = (*p*_*a*,*i*_, *o*_1_, … , *o*_*n*_) ∈ *X*_*g*_ *, with* *p*_*a*,*i*_ = (*w*_*a*,*i*_, *h*_*a*,*i*_) ∈ *A*_*r*,*f*_ *, and* *x*_*j*_ = (*p*_*a*,*j*_, *o*_1_, … , *o*_*n*_) ∈ *X*_*g*_ *with* *p*_*a*,*j*_ = (*w*_*a*,*j*_, *h*_*a*,*j*_) ∈ *A*_*r*,*f*_, *there exists* $t\in \Sigma_{g}^{*}$ *, such that* *δ*_*g*_(*x*_*i*_, *t*) = *x*_*j*_ *and* $t^{\prime }\in \Sigma_{g}^{*}$ *such that* *δ*_*g*_(*x*_*j*_, *t*′) = *x*_*i.*_
*A4*) *A connection between two rooms in MG* (*e.g., room* *A* *is connected to room* *B*) *means that considering that the door that connects the two rooms is open, then, for all objects, for any legal object configuration* *c*_*i*_ *, there exists a pair of states* *x*_*i*_, *x*_*j*_ ∈ *X*_*g*_ *, where* $p_{a,i}=\left(w_{a,i},h_{a,i}\right)\in A_{R_{A},f}$ *and* $p_{a,j}=\left(w_{a,j},h_{a,j}\right)\in A_{R_{B},f}$ *and a path* $t\in \Sigma_{g}^{*}$ *, s.t.* *δ*_*g*_(*x*_*i*_, *t*) = *x*_*j*_ *.*

We can easily create simulated environments in MG that follow the aforementioned assumptions. Specifically, for A3, we simply neither want to have isolated areas of free cells of the same room^5^ nor do we want, with A4, to obstruct the transition between rooms.

### 2.4 Abstraction in TextWorld

The finite abstraction of the real workspace can be expressed in TW by incorporating the information from A1 and by defining an appropriate environment state and transition function. We can replace TW abstraction with any other abstraction of the simulated environment which can be modeled as a DFA.

Definition 5 *Environment state in TextWorld* (Côté et al. 2019)*. A game state* *s* ∈ *S* *is a set of true logical propositions* *s* = (*p*_1_, … , *p*_|*s*|_). *For example, a logical proposition is* p≔ *“The apple is inside the fridge.” For illustration purposes, we define the environment state using a running example.*

**FIGURE 2:**
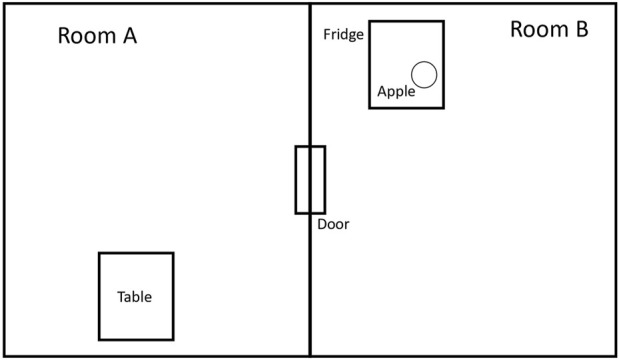
Overview of the workspace, where labels are assigned to important elements of the environment.

Example 1 *We have the following example, which is shown in* Figure 2. *In our example, we have*

1. *The set of different rooms* *R* = {*R*_*A*_, *R*_*B*_};
2. *The set of movable objects* *M* = {*apple*};
3. *The set of containers* *C* = {*fridge*, *door*}; *and*
4. *The set of objects that support other objects* *Sup* = {*table*}.

*The initial state is* *s*_0_ = (*p*_1_, *p*_2_, *p*_3_, *p*_4_, *p*_5_, *p*_6_)*, where* *p*_1_, *p*_2_, *p*_3_, *p*_4_, *p*_5_, and *p*_6_ *are the following logical propositions:* $p_{1}≔$ *“The fridge is at Room* *B* *,”* $p_{2}≔$ *“The fridge is closed,”* $p_{3}≔$ *“The agent is at Room* *A* *″,* $p_{4}≔$ *“The door is closed,”* $p_{5}≔$ *“The table is at Room* *A* *,” and* *p*_6_≔ *“The apple is inside the fridge.”* *We revisit now the set of logical rules provided by* Côté et al. (2019) (*i.e., the existing knowledge base* ^6^) *for environments that belong to Theme:“Home*.*”*

Definition 6 Knowledge base. For *c* ∈ *C*, *r* ∈ *R*, *sup* ∈ *Sup*, and *m* ∈ *M*, we have the following set of logical rules for our problem:

i.*open*(*c*)≔{*at*(*P*, *r*) ∧ *at*(*c*, *r*) ∧ *closed*(*c*)} ⇒ *opened*(*c*)
ii. *close*(*c*)≔{*at*(*P*, *r*) ∧ *at*(*c*, *r*) ∧ *opened*(*c*)} ⇒ *closed*(*c*)
iii. *take*(*m*, *c*)≔{*at*(*P*, *r*) ∧ *at*(*c*, *r*) ∧ *in*(*m*, *c*)} ⇒ *in*(*m*, *I*)
iv. *take*(*m*, *sup*)≔{*at*(*P*, *r*) ∧ *at*(*sup*, *r*) ∧ *on*(*m*, *sup*)} ⇒ *in*(*m*, *I*)
v.*put*(*m*, *sup*)≔{*at*(*P*, *r*) ∧ *at*(*c*, *r*) ∧ *open*(*c*) ∧ *in*(*m*, *I*)} ⇒ *on*(*m*, *sup*)
vi.*insert*(*m*, *c*)≔{*at*(*P*, *r*) ∧ *at*(*sup*, *r*) ∧ *in*(*m*, *I*)} ⇒ *in*(*m*, *c*)

Example continued: The initial state (*s*_0_ = (*p*_1_, *p*_2_, *p*_3_, *p*_4_, *p*_5_)) is expressed using the following logical propositions: *p*_1_≔*at*(*fridge*, *R*_*B*_), *p*_2_≔*closed*(*fridge*), *p*_3_≔*at*(*P*, *R*_*A*_), *p*_4_≔*closed*(*door*), *p*_5_≔*at*(*table*, *R*_*A*_), and *p*_6_≔*in*(*apple*, *fridge*).

The abstraction in TW can be expressed as a DFA *TextWorld* = (*S*, *Σ*, *δ*_*T*_, *x*_0,*T*_).

Definition 7 *For DFA* *TextWorld* = (*S*, *Σ*, *δ*_*T*_, *x*_0,*T*_)*, we have*

i.*The set of environment states in TW* *s* ∈ *S* (*Definition 4*).
ii. *The set of actions,* Σ = Σ_*M*_ ∪ Σ_*N*_ *, where*
*–*Σ_*M*_ = {*open*(*c*_1_), *open*(*c*_2_), …, *take*(*f*_1_, *c*_1_), …, } *is the set of manipulation actions for* *TW* *and*
*–*Σ_*N*_ = {*go west*, *go east*, *go north*, *go south*} *is the set of navigation actions for* *TW.*
iii. *The transition function* *δ*_*T*_(*x*, *σ*) = *x*′*, for* *x*, *x*′ ∈ *S* *, and* *σ* ∈ Σ *is described using the aforementioned logical rules that are stored in the knowledge base and define what is possible in the game.*
iv. *The initial state* *x*_0,*T*_ = *s*_0_.

It is noteworthy that in this environment, the location of each object is the room the object is in and not a set of grid coordinates like the previous state. In this way, the constructed TW environment abstracts the concept of room distance found in MG and greatly simplifies the problem. Sub-tasks such as “go to the bedroom” can be rewarded in MG only after the agent reaches that room and not as the agent approaches it. In other words, actions that do not reach the sub-task state, such as movement, do not receive a reward.

### 2.5 Multiple Choice User Interface

We consider user-defined tasks in an MCUI. In our framework, a user defines the task specification as a triplet *task* = (*obj*_1_, *obj*_2_, *loc*), where *obj*_1_ ∈ *M* is an object that can be picked up/dropped off and carried by a mobile robotic platform (e.g., a bottle of water), *obj*_2_ ∈ *Sup* is a support object (e.g., a table) on/at which the movable object (defined in 1) can be placed in *loc* ∈ *R*, which is a location which is the destination for the movable object (*obj*_1_). The information from MCUI (i.e., *task*) is used to identify all goal states in a reinforcement learning problem expressed in TW.

Definition 8 *The set of goal states for a task specification* *task* = (*obj*_1_, *obj*_2_, *loc*)*, where* *obj*_1_ ∈ *M* *,* *obj*_2_ ∈ *Sup* *, and* *loc* ∈ *R* *, is* *S*_*task*_ = {*s* = (*p*_1_, *p*_2_, … , *p*_|*s*|_) ∈ *S*: *∃*[*p*_*i*_≔*at*(*P*, *loc*)] ∧ [*p*_*j*_≔*on*(*obj*_1_, *obj*_2_)] ∈ *s*}. *The reward for all* *s* ∈ *S*_*task*_ *is* $R\left(s\right)=100+\frac{100}{n}$ *, where* *n* *is the number of the total steps needed for the agent to reach state* *s*. *We attempt to find the shortest path that leads to a goal state.*

### 2.6 Reinforcement learning problem formulated in TextWorld (RL–TextWorld)

Essentially, a reinforcement learning agent in TW explores the DFA *TextWorld* = (*S*, *Σ*, *δ*_*T*_, *x*_0,*T*_).

The RL problem is terminated when the RL agent reaches a state *s* ∈ *S*_*task*_. The sequence of actions that leads the agent to that state is *σ*[1]*σ*[2]…*σ*[*n*], and the corresponding state trajectory is *s*[0]*s*[1], …, *s*[*n*], where *s*[0] = *s*_0_ and *s*[*n*] = *s*, and for *i* ∈ {1, *n*}, we have *s*[*i*] = *δ*_*T*_(*s*[*i* − 1], *σ*[*i*]).

Example 1 *We specify the task “Put the apple on the table,” which is formally defined as the triplet* *task* = (*apple*, *table*, *RoomA*). *Solving the RL problem in TextWorld* (*see* Section 4.1), *we obtain the following action sequence:* *σ* ^6^ = *σ*[1]*σ*[2]*σ*[3]*σ*[4]*σ*[5]*σ*[6]*, where* *σ*[1] = *open*(*door*)*,* *σ*[2] = *go east* *,* *σ*[3] = *open*(*fridge*)*,* *σ*[4] = *take*(*apple*, *fridge*)*,* *σ*[5] = *go west* *, and* *σ*[6] = *put*(*apple*, *table*).

### 2.7 Reinforcement learning problem formulated in MiniGrid (RL–MiniGrid)

*In a simulated environment captured by DFA* *G* (*introduced in* Section 2.3)*, we formulate the RL–MiniGrid problem, which is guided by a dense reward function* *R*_*d*_ *as follows:*

a) *We automatically merge sequentially executed navigation tasks, with a successive manipulation task, producing a new task sequence* *σ*′[1]*σ*′[2]…*σ*′[*n*′]*, where* *n*′ ≤ *n*. *For example,* *σ*[1]*σ*[2] = (*go east*) (*open*(*fridge*)) *is merged to* *σ*′[1] = *open*(*fridge*).
b) *For an action* *σ*′*, a state* $x^{p}=\left(w_{a}^{p},h_{a}^{p},o_{1}^{p},\ldots ,o_{k}^{p}\right)\in X_{G}$ (*the previous state, before the execution of* *σ*′) *and state* $x^{c}=\left(w_{a}^{c},h_{a}^{c},o_{1}^{c},\ldots ,o_{k}^{c}\right)\in X_{G}$ (*the current state, after the execution of* *σ*′), *we define the following set of rules* *Rules* = {R1.1, *R*1.2, *R*2.1, *R*2.2, *R*2.3}*:*
  1. *For a container object* *o*_*i*_ ∈ *C*, *we have*
    1) R1.1 *which is satisfied if* (*σ*′ = *open*(*o*_*i*_)) ∧ (*prop* ^*p*^(*i*) = 0) ∧ (*prop* ^*c*^(*i*) = 1);
    2) R1.2 *which is satisfied if* (*σ*′ = *close*(*o*_*i*_)) ∧ (*prop* ^*p*^(*i*) = 1) ∧ (*prop* ^*c*^(*i*) = 0).
  2. *For a movable object* *o*_*m*_ ∈ *M* *, a container or support object* *o*_*j*_ ∈ *C* ∪ *Sup* *, a container object* *o*_*i*_ ∈ *C* *, and a support object* *o*_*s*_ ∈ *Sup*, *we have*
    1) R2.1 *which is satisfied if* (*σ*′ = *take*(*o*_*m*_, *o*_*j*_)) ∧ (*carry* ^*p*^(*m*) = 0) ∧ (*carry* ^*c*^(*m*) = 1);
    2) R2.2 *which is satisfied if* $\left(\sigma^{\prime }=put\left(o_{m},o_{s}\right)\right)\wedge \left(carry^{p}\left(m\right)=1\right)\wedge \left(carry^{c}\left(m\right)=0\right)\wedge \left(p_{m}^{c}=p_{s}^{c}\right);$

    3) R2.3 *which is satisfied if* $\left(\sigma^{\prime }=insert\left(o_{m},o_{i}\right)\right)\wedge \left(carry^{p}\left(m\right)=1\right)\wedge \left(carry^{c}\left(m\right)=0\right)\wedge \left(p_{m}^{c}=p_{i}^{c}\right).$

c) *We compute the dense reward function, as shown in* Algorithm 1. *In particular, we are inspired by ideas used by* Mataric (1994) *applied to a problem with multiple sub-goals. In our case, we use the number of remaining sub-tasks as a metric to measure the progress toward the user-defined task.*

### 2.8 Proof that the hierarchical RL algorithm can derive solutions

Theorem 1 If we have a DFA *G* in MG, its abstraction is expressed as a DFA-*TW*, and the assumptions A1–A4 hold, then we prove that RL-TW does not restrict the RL-MG from deriving solutions.

Proof 1 The following lemmas are based on A1–A4:

Lemma 1 i) A manipulation action that involves an interaction with *obj*_*i*_ changes only configuration *c*_*i*_ (of *obj*_*i*_) and ii) a navigation action changes only the position of the agent (*p*_*a*_) and the position of the objects that the agent is carrying.

Lemma 2 If room *A* is connected with room *B* and the door that connects them is open, then the agent can reach any free cell in room *B* $\left(A_{R_{B},f}\right)$ starting from any free cell in room *A* $\left(A_{R_{A},f}\right)$ , executing only navigation actions and *vice versa*.

Lemma 3 The execution of a manipulation action (Σ_*M*,*g*_) is required for the satisfaction of a *rule*.

Lemma 4 Between two successively satisfied *rules* ∈ *Rules* (*Rule*[*k*] and *Rule*[*k* + 1]), we do not need to execute any other manipulation action.

Lemma 5 If we are at a state where *Rule*[*k*] is satisfied and *Rule*[*k* + 1] is not satisfied yet, then we can always reach a state where *Rule*[*k* + 1] can be satisfied by executing only navigation actions (Σ_*N*,*g*_).

A sequence *σ*′[1]…*σ*′[*n*′] (sequentially satisfied sub-goals) is given as input to the RL-MG problem to restrict its solutions to only those that sequentially satisfy the rules *Rule*[1]*Rule*[2]…*Rule*[*n*′], where *Rule*[*k*] ∈ {*R*1.1, *R*1.2, *R*2.1, *R*2.2, *R*2.3} corresponds to the conditions satisfied for *σ*′[*k*].

Lemma 1 allows us to argue that manipulation and navigation actions are completely disentangled, and they affect different properties of the agent and/or objects present in the environment. From Lemmas 3 and 4, we argue that the sequence of the manipulation actions that we need to execute is the one that sequentially fulfills the conditions of *Rule*[1]*Rule*[2]…*Rule*[*n*′]. Moreover, from Lemma 5 (which is based on Lemma 2), we also argue that sequences of only navigation actions are executed in-between the satisfaction of successive rules. Concluding our proof, it is easy to argue that any sequence of sub-goals (from RL-TW) that is provided as input to the RL-MG problem does not restrict the problem from deriving to solutions^7^

Theorem 1 can be easily generalized to be true to all other environments, which can be expressed as DFAs. The analysis can be done similarly as it is illustrated in this proof for TW and MG environments.

## 3 Results

Our proposed methodology is applied to the running example. The results are shown in Sections 3.1, 3.2. Next, we run the previous example with the same hyperparameters at different levels of difficulty. These results demonstrate the increasing need of dense rewarding even in such relatively simple environments.

### 3.1 Running example: TextWorld framework (RL-TW)

**FIGURE 3:**
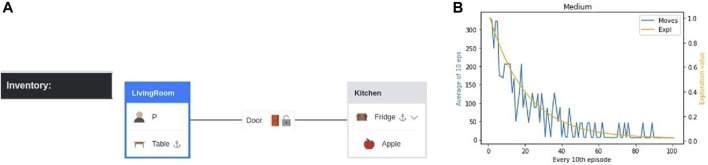
**(A)** The player/agent is set into room A, and a door is added to the corridor connecting the rooms A and B. Supporter and container items are added to each room, and the remaining items are added on the floor, on top of supporter items, or inside container items. The graphical representation given here is not part of the game, which is text-based. **(B)** Training with the Q-learning agent in TW. X-axis-left (blue) is the average number of actions taken in the span of every 10 episodes in the *y*-axis. X-axis-right (orange) is the exploration that indicates the probability the action taken is random instead of policy-based and decreases with every iteration (as the agent learns).

**FIGURE 4:**
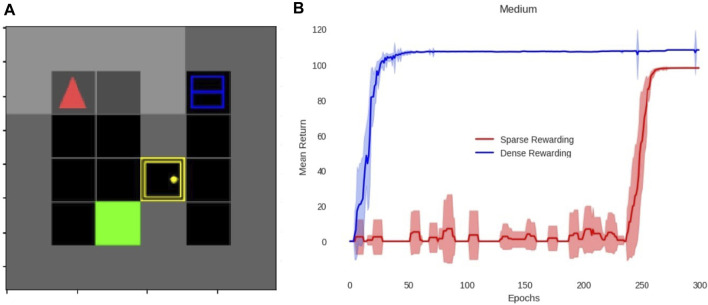
**(A)** Simulation environment of MG for the running example. The green square is the table, the yellow block is the door, the blue container is the fridge, which has a red ball inside (i.e., the apple), and the red arrow is the player. The black blocks are empty cells, the gray blocks are walls, and the key (only present in the hard environment shown in Figure 6) is self-evident. **(B)** Results of MiniGrid training. The *x*-axis is the average return of 16 episodes every run for 300 runs (*y*-axis), which we call epochs. The results with (blue) and without (red) our method are shown. The shaded region around the lines indicates the standard deviation. The maximum return of sparse-rewarding reaches lower than that of dense-rewarding since it lacks the sub-task rewards.

First, we construct the simulation of the example (Figure 2) in the MG environment (Figure 4) with the aforementioned assumptions (see A1–A4). Then, we construct the abstraction in TW, as shown in Figure 3. The user interacts with a text-based interface (Section 2.5). Specifically, we ask the user to input the desired movable object in the desired location and on the desired supporter object. In our example, the movable object is “Apple,” the location is “Room A,” and the supporter object is “Table.” We continue by automatically constructing the goal state, i.e., “place apple on table” while situated in room A. Training in TW via a Q-learning algorithm (Watkins and Dayan, 1992) is run for a total of 1,000 episodes with a maximum of 400 steps each. In each step, the agent takes an action, and the episode ends whenever the agent reaches the goal or when the maximum number of steps is reached. At the end of the training, the agent is evaluated by being tested in the same environment (reliant only on its policy, unaffected by the exploration), and the following actions are produced:

Open door → go east → open fridge → take apple from fridge → go west → put apple on table.

**FIGURE 5:**
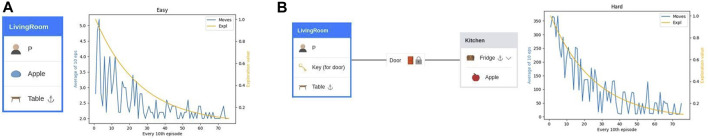
**(A)** Environment and training for the easy version of the example in TW and **(B)** environment and training for the hard version of the example in TW.

This is the optimal solution, meaning that there is no shorter task path than this. This result shows that TW efficiently decomposed our example command into sub-tasks. The illustrated example is the medium-difficulty environment. For the purpose of showing the limitations of sparse rewarding, we test our method in three different levels of difficulty (easy, medium, and hard). The other two are shown in Section 3.3. Although training here requires a high number of iterations, the training time does not surpass 1 min. The spikes observed during training are caused by the decaying exploration value which reaches a minimum of 0.05, which corresponds to a 5% chance that the action taken is random. However, during evaluation, when the agent takes actions based on its policy, the actions are the minimum required. The same is true for the figures of the easy and hard difficulties (Figure 5).

### 3.2 Running example: MiniGrid framework (RL-MG)

Reward function in MG is designed using Algorithm 1 (exploiting TW training). Training in MG uses the PPO algorithm (Schulman et al., 2017). The MiniGrid training scripts allow for the use of the A2C algorithm (Mnih et al., 2016) as well. Exactly because we wanted to highlight that our approach is decoupled from the underlying RL algorithm, we did not perform any particular performance analysis on the available algorithms. On the contrary, we utilized the “go to” option for the RL problems with discrete actions, which is the PPO approach, without modifying a thing. Of course, and if needed by the actual robotic application, one could utilize different algorithms or perform hyperparameter tuning to acquire better (more tailored to the problem at hand) solutions. We also included a LSTM network (Hochreiter and Schmidhuber, 1997) in the agent neural network to introduce memory. Specifically, during training, the agent remembers the last eight actions performed. Training occurs over 16 environments running in parallel for 300 episodes each. The environments differ only in the randomness of their exploration. Each episode lasts for a maximum of 128 steps or until the agent reaches the ultimate goal (i.e., the user command). We plot the average return of the 16 environments every episode. More details regarding the hyperparameters of training are given in Appendix A.

Algorithm 1 Computation of the dense reward function *R*_*d*_-subroutine.

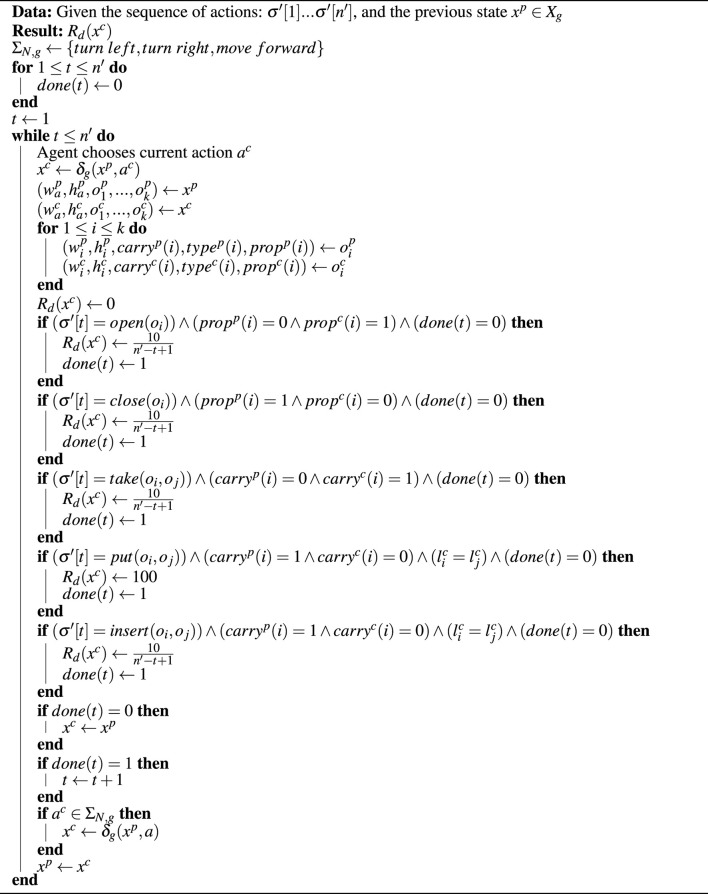

**FIGURE 6:**
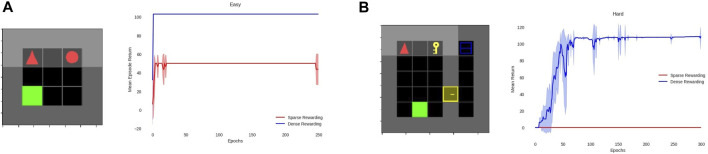
**(A)** Environment and training for the easy version of the example in MG and **(B)** environment and training for the hard version of the example in MG.

Figure 4B shows the result of the medium-difficulty environment. This environment has a total of four sub-tasks due to the sub-tasks “go east” and “go west” being fused with manipulation actions, as mentioned in Section 2.7. This result shows that the agent with our method greatly outperforms the agent without it, for the running example. The difference in the peak return in Figure 4B and Figure 6 between the two lines is due to sub-tasks adding more rewards with our method and should not be confused with performance. Instead, performance should be judged based on the number of epochs until each agent reaches saturation (i.e., learns the solution).

### 3.3 Additional runs

In order to better test results with and without our approach, we examined two more iterations of the previous example. The first one is the easy version, and the second, the hard version. The easy environment decreases the number of sub-tasks to just two, while the hard environment increases them to five by adding the “take key”, “open door” and “open” sub-tasks (unlocking the door is assumed to happen within the “open door” sub-task) and also increasing the size of the overral grid to 7 × 7. Again, as in the example, we show the TW environment and training (Figure 5) and MG environment and training (Figure 6) but now for the a) easy and b) hard difficulties.

The hard environment shown in Figure 6B is one sub-task harder and a little larger than the medium environment in Figure 4A. As shown in the graphs, this difference is enough for the PPO agent to reach its limits and never reach the goal. These versions show that in very simplistic environments, our method does not provide substantial improvement, but in the case of a more complex environment (still far less complex than the real world), it is crucial. The standard deviation spikes that appear at the start of MG training (Figure 6; Figure 4) are due to the 16 environments running in parallel that converge at different times as a result of the randomness in exploration. On the other hand, the lack of spikes during the middle and end of the training indicates that all environments converged to the same solution, which we checked to be the optimal.

Training also presented some challenges. We chose Q-learning since it can be much faster in simple problems. Despite this, the trade-off is that as the number of feasible states and feasible actions increases, the size of the Q-matrix also increases proportionally which, in turn, increases the time it takes for the weights to be updated and the overall training time. It became painstakingly clear that Q-learning without a neural network will not suffice in more complex environments. In an effort to reach convergence, we experimented with negative rewards provided to irrelevant actions. Our code still has that option, but after some fine-tuning of hyperparameters, we concluded that this was not necessary.

## 4 Discussion

Section 4.1 summarizes the main points of our work. Section 4.2 discusses the shortcomings of our work; Section 4.3 presents the implications, while Section 4.4 discusses possible directions we consider worthwhile pursuing.

### 4.1 Conclusion

It is self-evident that even in such an elementary and minimal environment compared to the real world, home agents require guidance from dense reward functions to learn to carry out complex tasks. Task decomposition is an easy-to-use approach for introducing those dense rewards. We formulated a method that can be used to improve training in embodied AI environments by harnessing the task decomposition capabilities of TW, proved it can provide optimal solutions in our framework, and demonstrated its efficacy in MG. A shortcoming of the proposed method is that, for every simulation environment, a TW environment must be built manually, which can be quite arduous.

### 4.2 Limitations

HRL algorithms have shown to speed up many offline planning algorithms (Sacerdoti, 1974; Dean and Lin, 1995), where the dynamics of the environment are known in advance. However, in the real world, pre-existing knowledge about the environment is not always possible. Even worse, many times, the environment is dynamic. Current embodied AI simulators do not take into account dynamic variables and consider the environment static and, sometimes, known. This is the case in our setup as well. Every time the environment changes, we would need to retrain our agent. Theoretically, trained in large, diverse datasets, the agents could develop a problem-solving policy that handles all possible environments. Even if we consider the real environment to be a white box, another limitation is that we currently need to manually construct it inside the simulator and then inside the levels of abstraction.

Yet another limitation is the assumption of deterministic setups (also commonly found in simulations). In other words, instead of specific actions leading to specific states (based on the current state), they could lead to different states in a stochastic manner. For example, in real-world scenarios, a robotic agent might fail to grasp the bottle of water due to noise or errors, leading to the same state it was in previously instead of the state with the bottle inside its inventory. Theoretically speaking, we can extend our definition to support stochasticity.

### 4.3 Implications

For robotic tasks specifically, HRL is a powerful tool to simplify the complete challenge. In many applications, researchers break down the challenges to distinct tasks that are easier to manage, removing the need for a global RL agent to disentangle them on its own. If the vision of a complete embodied AI agent is to be realized, it will encompass multiple modules, each handling a different task and a robust training dataset. The task decomposition itself is important since it can become complicated and arduous if done manually, but without proper rewards for each sub-task, the modules will not be trained adequately. Therefore, our method (with the right adjustments) that decomposes the problem in order to assign rewards can prove to be a big boost to any attempt at an embodied AI agent. For a broader use, we would need to define a general deterministic finite automaton (or multiple with slight variations) that applies to existing popular simulators and then, a compatible code that would automatically apply the resulting sub-task reward from TW to that simulator. Lastly, for handling dynamic cases, we would need to extend to an NFA.

Moreover, our experiments could be used in reference to emphasize the adverse performance of sparse rewards even in simplistic problems or for demonstrating TW task decomposition capabilities. The current work can also be adopted, as is, for finding a task plan and then finding more fine-grained navigation actions. In other words, a more advanced simulation can be placed on top of our work which will benefit from sub-tasks or sub-actions of TW and MG, respectively.

### 4.4 Future work

For future work, we aim to make the process of constructing an abstraction of the environment automatic. Additionally, our lowest level of hierarchy might be far from being characterized realistic, but replacing it with a simulator that allows more low-level robotic actions such as Habitat (Manolis Savva* et al. (2019)) only requires the manual abstraction of the environment and adapting to the reward-assigning module. Alternatively, MG could stand as the second level of hierarchy that helps with the enrichment of the third lower-level environment for additional navigation actions. Another direction that we are interested to pursue is using temporal logic to specify the complex task and design the reward function. Extensions of this work will also explore more general setups.

The upcoming Habitat challenge of 2023 (Yenamandra et al., 2023a) has Open-Vocabulary Mobile Manipulation (OVMM) tasks (the goal is a text description instead of coordinates), and they access the partial success of sub-tasks, which means it is possible that they can provide rewards for those sub-tasks as well. However, these are limited to the tasks quote-on-quote “1) finding the target object on a start receptacle, 2) grasping the object, 3) finding the goal receptacle, and 4) placing the object on the goal receptacle (full success).” Our approach could enhance the sub-tasks even more by introducing sub-tasks like “go to the {room}” where {room} can be replaced by existing rooms (i.e., bedroom, living room, and toilet). Moreover, MG could introduce navigation sub-tasks such as “go forward” and “turn right.” We intend to adapt our method to the environments of this and future competitions. By replicating the winning implementations, we can more convincingly showcase training speedups and, consecutively, evaluation performance improvement.

## Data Availability

Publicly available datasets were analyzed in this study. These data can be found at: https://github.com/AthanasiosPetsanis/DiplomaClone.

## Footnotes

1 This is a simplification. The NN we mention could comprise different NNs and other modules, but ultimately, this will be the box (black/gray/white) that is trained and is responsible for mapping the commands to robotic actuation.

2 To be exact, there are verbs that TextWorld does not recognize and will inform the player in such a case (e.g., “grab the water bottle” results in “This is not a verb I recognize.” However, since the knowledge base is extendable, one can add the verb “grab” as an alternative to “pick up.”

3
https://github.com/AthanasiosPetsanis/DiplomaClone

4
https://github.com/AthanasiosPetsanis/DiplomaClone/blob/main/Main.ipynb

5 Note that only support objects or walls can cause a possible isolation, and the agent can move to a free cell in which a movable object is placed (i.e., dropped).

6 The knowledge base can be easily extended by adding extra rules.

7 The more general case of nondeterministic models, like general MDPs, is left for a future work.

## Appendix A: Hyperparameters

For the purposes of reproducibility, in Tables A1, A2, we list the hyperparameters for the RL agents of TW and MG, respectively. Alternatively, one can reproduce the results by running the notebook file (found at: https://github.com/AthanasiosPetsanis/DiplomaClone/blob/main/Main.ipynb) in Google Colab.

**TABLE A1:** TW training hyperparameters.

Hyperparameter	Value
max_epochs	5
max_eps	150
min_expl	0.05
expl_decay_rate	0.004
gamma	0.9
user_input	“put apple on table”

**TABLE A2:** MG training hyperparameters.

Hyperparameter	Value
frames	614,400
frames_per_proc	128
graph_points	300
recurrence	8
seed	1
procs	16
save_interval	10
log_interval	1

Starting with Table A1, the goal state is defined by the user_input, which is the same for all levels of difficulty and equal to the string “put apple on table.” The rest of the variables were tuned empirically in order to see convergence in all TW training examples. Here, we use the term “epochs” for nothing more than the total of 150 steps. Therefore, five epochs of the 150 steps mean a total of 750 steps, which was the minimum necessary number of steps in order for the algorithm to achieve the optimal result. The value 0.004 of the exploration decay rate (expl_decay_rate) ensured that by the end of the training for any of the tested environments, the agent reached the minimum exploration value (min_expl) of 0.05 and, therefore, relied mostly on the learned policy. The min_expl value was not chosen to be 0 in order to allow deviations from the learned policy and potentially discover better routes. This is a common practice during training RL agents. It is also common to use a high value for the discount variable gamma but less than 1 since the future reward is less valuable than the present reward.

For Table A2, frames are the total number of steps for the experiment defined *frames* = *graph*_*points* ⋅ *procs* ⋅ *frames*_*per*_*proc*, where *graph*_*points* is the number of points in the MG-training graph, procs is the number of processes (i.e., environments) running in parallel, and *frames*_*per*_*proc* are the frames per process before updating. We empirically chose *graph*_*points* to be 300, while the default values of procs and frames_per_proc are 16 and 128, respectively. Recurrence is the number of times the step gradient is backpropagated (default 1). If >1 , an LSTM is added to the model to introduce memory. Seed, as usual, determines the pseudo-random number generation of the code. Save_interval indicates how many graph points the results are saved. Log_interval indicates how many graph points the results will be displayed on the terminal. More details can be found in the code: rl-starter-files.
